# Unsupervised Spectral-Spatial Feature Selection-Based Camouflaged Object Detection Using VNIR Hyperspectral Camera

**DOI:** 10.1155/2015/834635

**Published:** 2015-03-23

**Authors:** Sungho Kim

**Affiliations:** Yeungnam University, Gyeongsan, Gyeongbuk 712-749, Republic of Korea

## Abstract

The detection of camouflaged objects is important for industrial inspection, medical diagnoses, and military applications. Conventional supervised learning methods for hyperspectral images can be a feasible solution. Such approaches, however, require a priori information of a camouflaged object and background. This letter proposes a fully autonomous feature selection and camouflaged object detection method based on the online analysis of spectral and spatial features. The statistical distance metric can generate candidate feature bands and further analysis of the entropy-based spatial grouping property can trim the useless feature bands. Camouflaged objects can be detected better with less computational complexity by optical spectral-spatial feature analysis.

## 1. Introduction

The development of an image sensor and optical dispersion technology has made it possible to capture hyperspectral image data with lower prices, such as SPECIM or Honeywell products [1]. Therefore, it is possible to inspect and develop algorithms from acquired hyperspectral images instead of a public database of remote hyperspectral images, such as AVIRIS and Hyperion [2]. Currently, hyperspectral images are used frequently in a range of areas to detect important parts such as cavities in medical applications and crime in forensic applications [1, 3].

Although spectral information can be useful for discriminating camouflaged or abnormal regions, the high dimensionality of the hyperspectral data leads to a huge increase in computational time, and the highly correlated bands contain a degree of redundancy, which might have a negative impact on detection. For example, if a single scan of a hyperspectral cube contains 1,392 pixels (samples), 1,000 pixels (scan length), and 1,040 (bands) with a 2 bytes A/D resolution, the total data size was approximately 3 GBytes. This is 600 times larger than the size of the full HD image (6 MBytes). Therefore, the key problem for the detection of hyperspectral abnormal regions is to reduce the computation complexity without degrading the detection accuracy. Therefore, reducing the dimensionality by the spectral band selection is often adopted to reduce computational cost and improve the accuracy.

Band selection can be achieved by supervised or unsupervised learning. The former requires a set of labeled training databases and produces the high accuracy of detection performance [4–7]. On the other hand, the number of training samples is limited in most hyperspectral applications. The latter requires no training images and detects abnormal regions directly from a test hypercube. Therefore, this study adopted the unsupervised learning-based band selection scheme for its convenience in automatic camouflaged or abnormal region detection. Recently, several studies proposed a range of band selection or elimination methods in unsupervised learning approaches focusing only on spectral analysis. Previous techniques of unsupervised band selection for hyperspectral images can be classified broadly into two categories: ranking-based methods [8, 9] and clustering-based methods [10, 11]. The ranking-based methods evaluate the relevance of each band independently to estimate the quality of the attributes depending on how well their values help classify the patterns using either the information divergences [9] or similarity-based band analysis [8]. On the other hand, clustering-based methods perform clustering on bands to group them according to their correlation and selects one band from each cluster representing the whole group, such as mutual information [11] or affinity propagation [10].

In the first stage of spectral feature analysis, a new statistical distance measure in the ranking-based method instead of the band clustering method was proposed due to the high computational complexity. Spectral analysis can generate candidate bands that maximize the statistical distance. In the second stage, an entropy-based measure was proposed to quantify the uncertainty of spatial segmentation. The bands that generate high entropy value (noisy spatial segmentation) can be reduced. Therefore, the first contribution is the proposition of a novel band selection method by considering both spectral and spatial analysis without prior knowledge. The second contribution is the automatic detection of a camouflaged or abnormal region without a training process. Therefore, the detected results can be obtained without human intervention if any kinds of hyperspectral test images are applied to the inspection system.  Section 2 introduces the proposed camouflaged target detection method using spectral-spatial feature analysis.  Section 3 validates the proposed method according to various band selection schemes and Section 4 concludes the study.

## 2. Proposed Camouflaged Object Detection Method

### 2.1. Overview of the Proposed Inspection System

**Figure 1:**
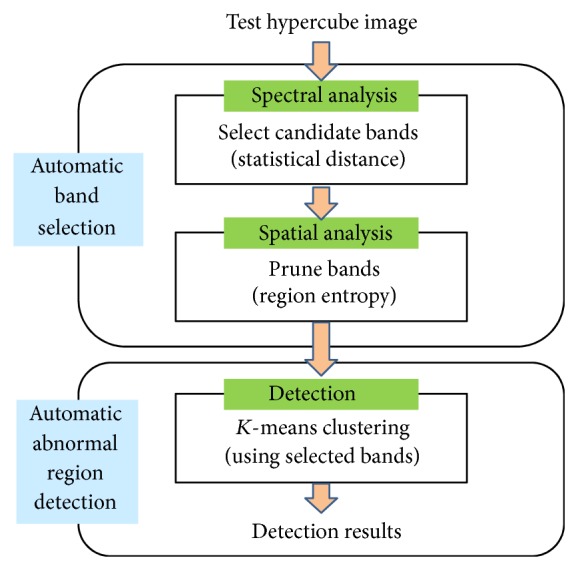
Overall inspection flow of the proposed system.

Figure 1 summarizes the overall flow of the proposed hyperspectral inspection system. Given a test hypercube image, the automatic band selection block is activated by consecutive spectral and spatial analysis. Statistical distance analysis of each spectral band generates a discriminating curve. The candidate spectral bands can be obtained through the local maxima of the curve. Segmented regions can be obtained using each band with cluster labels. The underlying assumption is that good band segments the input image into two regions: camouflaged and background regions. The number of segmented regions was quantized using entropy. Therefore, entropy can represent the complexity of regions. Based on entropy, the optimal bands are selected. The final detection results were obtained using *K*-means clustering with the selected bands.

### 2.2. Spectral-Spatial Analysis-Based Band Selection

#### 2.2.1. Hypercube Acquisition System (Table 1)

**Figure 2:**
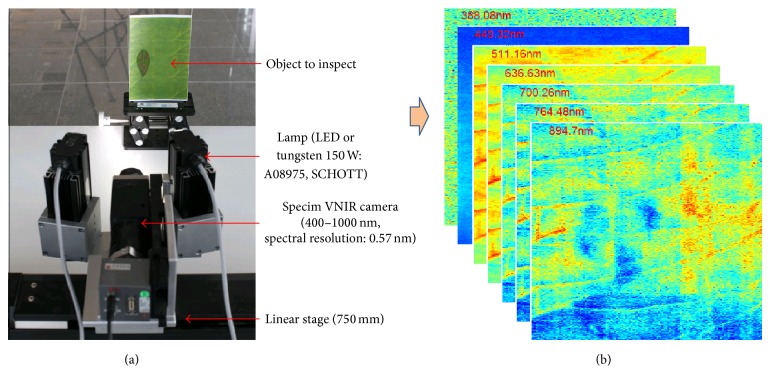
Hypercube: (a) VNIR hyperspectral image acquisition system and (b) sample spectral images.

The spectral image acquisition system consists of a SPECIM VNIR camera (PS-FW-11-V10E) mounted on a linear stage, LED, or halogen lamps and a target to inspect, as shown in Figure 2(a). Figure 2(b) shows sample spectral band images.

#### 2.2.2. Spectral Analysis

**Figure 3:**
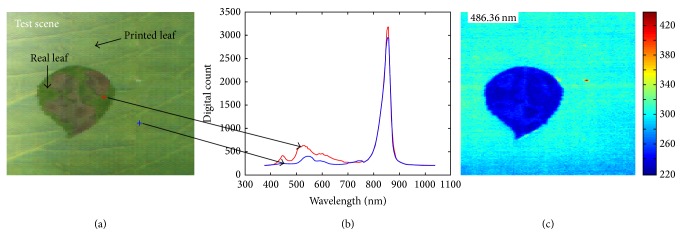
Spectral analysis: (a) test hypercube, (b) spectral profiles at the selected points, and (c) band image at 486.36 nm.

**Figure 4:**
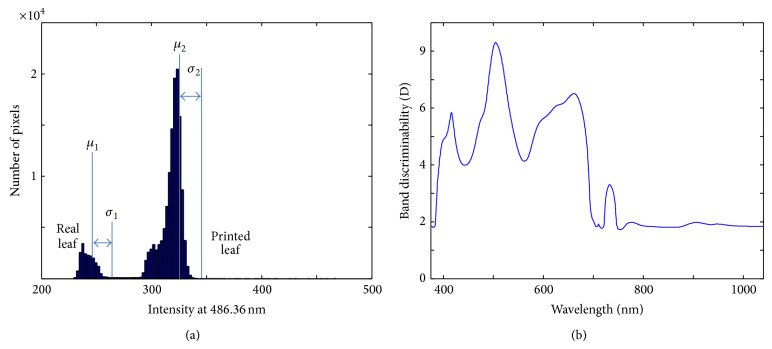
Spectral analysis: (a) test hypercube, (b) band image at 486.36 nm, (c) pixel distribution, and (d) proposed band discriminability graph.

A camouflaged object detection problem can be regarded as selecting suitable spectral bands that discriminate interesting region in normal background. The proposed statistical distance metric can be useful to generate candidate bands because a hypercube image provides enough samples (about millions) of spectral profiles and statistical distance can measure the distinctiveness of spectral bands, which leads to easy detection of camouflaged objects. For example, if a test hypercube consists of a real leaf and a printed leaf, the spectral profile and specific band image are obtained as shown in Figure 3. Statistical distance-based, candidate band selection is motivated by the observation of band image analysis, as shown in Figure 4(a). The distribution histogram can be made for a hypothesized band *b* [nm]. According to the distribution, two Gaussian distribution functions (foreground and background) parameterized from the means (*μ*_1_, *μ*_2_) and standard deviations (*σ*_1_, *σ*_2_) can be fitted. The class discriminability measure is defined as

(1) $$\begin{array}{c} D\left(b\right)=\frac{\left|\mu_{1}\left(b\right)-\mu_{2}\left(b\right)\right|}{\sigma_{1}\left(b\right)+\sigma_{2}\left(b\right)} \end{array}$$

By applying the aforementioned equation to each band, the band discriminability curve can be obtained according to the wavelength, as shown in Figure 4(b). The candidate bands can be selected by applying local maxima or global maxima to the curve.

#### 2.2.3. Spatial Analysis

**Figure 5:**
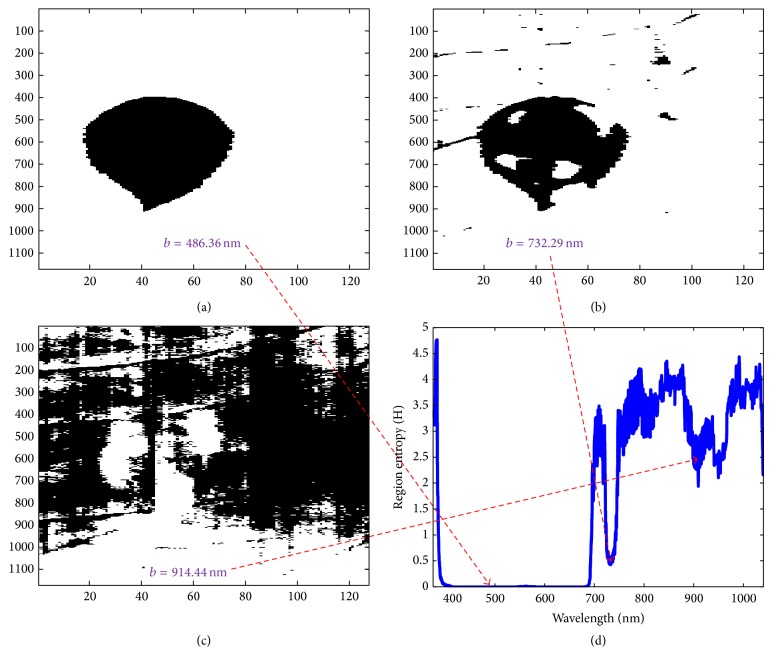
Spatial analysis: the large number of regions produces high region entropy score and two segmented regions produce lowest region entropy score.

*K*-means clustering can effectively cluster data using feature distance [12]. If *K*-means clustering (*K* = 2) with *b*th band image is performed, the discriminability value can be obtained as mentioned above. At the same time, a segmented image using the class labels in image space can be acquired. If a hypercube image has the size of samples (*S*) × scan length (*L*) × bands (*B*), the complexity of segmented regions at the *b*th band can be quantified using entropy. Entropy can measure the complexity of spatial region distribution. In the camouflaged object detection problem, the ideal number of regions is just two (foreground and background). Therefore, high entropy can represent large number of segmented regions. The region entropy is defined as

(2) $$\begin{array}{c} H\left(b\right)=-\overset{M}{\underset{i=1}{\sum }}p_{i}\left(b\right)\log p_{i}\left(b\right), \end{array}$$

where *p*_*i*_(*b*) is the probability of the pixels belonging to *i*th region. This is defined as *p*_*i*_(*b*) = *N*_*i*_(*b*)/(*S* × *L*). *M* denotes the total number of segmented regions and *N*_*i*_(*b*) denotes the number of pixels belonging to the *i*th region at the band image *b*. Ideally, the detection results consist of one abnormal region and the other background region. If the number of segmented region increases, the region entropy increases. Therefore, a threshold is applied for the region entropy to reduce the candidate bands that generate many small regions.  Figure 5 shows the region segmentation results according to the different region entropy values. The region entropy threshold around 1 is normally used.

## 3. Experimental Results

**Figure 6:**
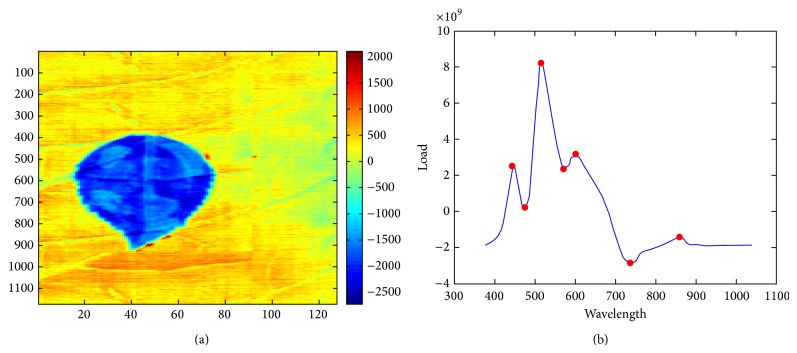
Baseline method of band selection by PCA [6]: (a) principal component: PC2 and (b) load function curve of PC2.

The proposed method was validated in terms of the band selection scheme using the same *K*-means clustering (unsupervised classifier). The baseline band selection method was principle components analysis (PCA), which is an effective data reduction technique that is used frequently in hyperspectral data analysis [6]. In PCA, a human manually selects a principle component (i.e., PC2 as shown in Figure 6(a)) that visualizes the abnormal region clearly. The optimal set of bands can be selected using the local maxima/minima from the loading curve of PC2 as shown in Figure 6(b).

**Figure 7:**
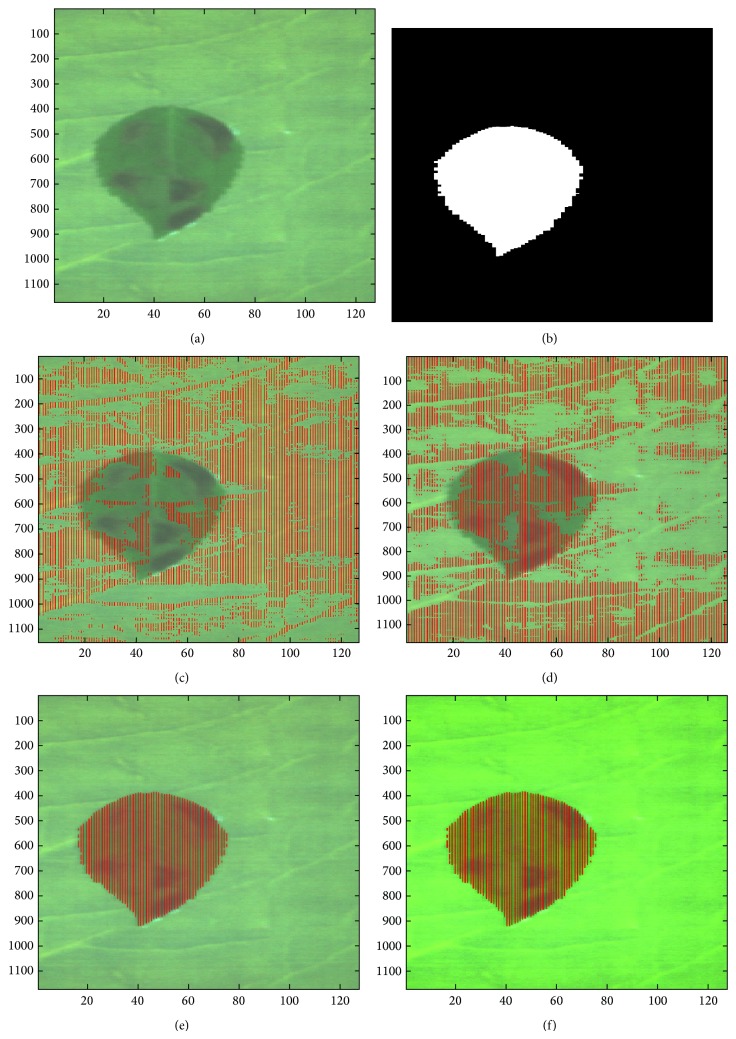
Abnormal region detection results: (a) test leaf image, (b) ground truth, (c) PCA method, (d) spectral profile, (e) Proposed 1 band selection by spectral analysis, and (f) Proposed 2 band selection by spectral-spatial analysis.

As a second baseline method, the entire spectrum curve, where all the bands are selected, is used [13]. These conventional methods are compared with the proposed band selection methods, such as band selection by spectral analysis (Proposed 1) and by spectral + spatial analysis (Proposed 2). The detection rate (DR), false alarm rate (FAR), and the number of bands used for quantitative comparison are used.  Table 2 lists the overall performance comparison of the leaf database. PCA method selected 7 bands (447.4, 475.2, 517.3, 572.9, 600.6, 740.0, and 858.0). PCA and profile methods showed similar detection results with a high false alarm rate of 45%. The Proposed 1 method selected 9 bands (416.2, 503.7, 660.7, 732.3, 776.8, 905.2, 948.1, 1000.6, and 1027.3 nm) and showed 100% of DR with 0.008% of FAR. The Proposed 2 method with 4 selected bands (416.2, 503.7, 660.7, and 732.3 nm) showed the optimal performance with the fewest number of bands. Figures 7(c)–7(f) show the qualitative performance comparison results for a given test hypercube (Figure 7(a)) and a ground truth image (Figure 7(b)). The Proposed 2 method could detect the camouflaged region perfectly. In terms of detection time complexity, the Proposed 2 method took only 0.66 seconds which is 9.1 times faster than the PCA and 84.1 times faster than the profile. The space complexity is proportional to the number of bands. So, the Proposed 2 method occupies the smallest memory space.

**Figure 8:**
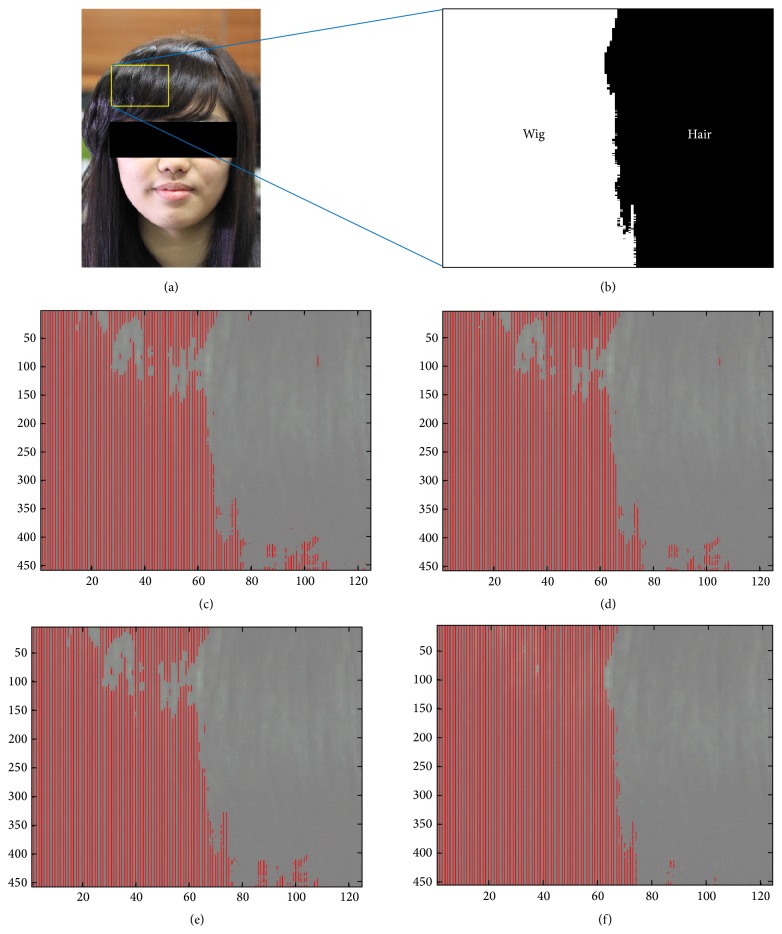
Abnormal region detection results: (a) test hair image, (b) ground truth, (c) PCA method, (d) spectral profile, (e) Proposed 1 band selection by spectral analysis, and (f) Proposed 2 band selection by spectral-spatial analysis.

Another test was conducted to validate the proposed method for the hair database, which consists of a wig and hair.  Table 3 summarizes the overall performance comparison for the leaf database. PCA, profile, and Proposed 1 methods showed similar detection results with a DR of 92% and FAR of 2~3%. The Proposed 2 method with 2 selected bands (945.5 and 1017.3 nm) showed the best performance with fewest number of bands. Figures 8(c)–8(f) show the qualitative performance comparison results for a given hair test image (Figure 8(a)) and a ground truth image (Figure 8(b)). As shown in Figure 8(a), detection errors occurred at the specular regions.

## 4. Conclusions

This letter proposed a novel band selection and abnormal region detection method in a completely unsupervised manner. From a test input hypercube, the proposed system generates candidate bands based on statistical distance analysis. The system removes bands that generate a number of region segments based on the region entropy measure. Experimental comparisons with the baseline methods validated the outperformance of the proposed method in terms of the detection rate and false alarm rate with a minimal number of bands for a real test set. The best abnormal region detection result with a few selected bands (2–4) can be obtained without human intervention in both band selection and detection.

## Figures and Tables

**Table 1:** Specifications of the hyperspectral image acquisition system.

Item	Specifications
Spectral range	400–1000 nm (VNIR)
Spectrograph	ImSpector V10E 30 *μ* slit, 2.8 nm spectral resolution
Camera	Kappa 1,392 × 1,040 pixels, 12 bits, 11 fps, FireWire interface
Lamp	150 W A08975, SCHOTT
Scanner	Linear stage, length 750 mm

**Table 2:** Comparison of abnormal region detection methods for the leaf database (DR: detection rate, FAR: false alarm rate, Proposed 1: spectral analysis, Proposed 2: spectral + spatial analysis).

Method	DR (%)	FAR (%)	Number of bands	Detection time (s)
PCA [6]	71.6	45.8	10	6.03
Profile [13]	73.5	45.2	1040	55.53
Proposed 1	100.0	0.0008	9	0.93
**Proposed 2**	**100.0 **	**0.0**	**4**	0.66

**Table 3:** Comparison of the abnormal region detection methods for the hair database.

Method	DR (%)	FAR (%)	Number of bands
PCA [6]	92.1	3.1	5
Profile [13]	92.8	2.2	1040
Proposed 1	92.4	2.4	8
**Proposed 2**	**99.9 **	**0.2**	**2**
